# Conductive hearing loss during development does not appreciably alter the sharpness of cochlear tuning

**DOI:** 10.1038/s41598-021-83115-1

**Published:** 2021-02-17

**Authors:** Yi Ye, Antje Ihlefeld, Merri J. Rosen

**Affiliations:** 1 Hearing Research Group, Department of Anatomy and Neurobiology, Northeast Ohio Medical University, 4209 State Route 44, Rootstown, OH 44272 USA; 2 Department of Biomedical Engineering, New Jersey Institute of Technology, Newark, NJ 07102 USA; 3 Brain Health Research Institute, Kent State University, Kent, OH 44272 USA

**Keywords:** Sensory processing, Neuronal development, Cochlea, Cortex

## Abstract

An increasing number of studies show that listeners often have difficulty hearing in situations with background noise, despite normal tuning curves in quiet. One potential source of this difficulty could be sensorineural changes in the auditory periphery (the ear). Signal in noise detection deficits also arise in animals raised with developmental conductive hearing loss (CHL), a manipulation that induces acoustic attenuation to model how sound deprivation changes the central auditory system. This model attributes perceptual deficits to central changes by assuming that CHL does not affect sensorineural elements in the periphery that could raise masked thresholds. However, because of efferent feedback, altering the auditory system could affect cochlear elements. Indeed, recent studies show that adult-onset CHL can cause cochlear synapse loss, potentially calling into question the assumption of an intact periphery in early-onset CHL. To resolve this issue, we tested the long-term peripheral effects of CHL via developmental bilateral malleus displacement. Using forward masking tuning curves, we compared peripheral tuning in animals raised with CHL *vs* age-matched controls. Using compound action potential measurements from the round window, we assessed inner hair cell synapse integrity. Results indicate that developmental CHL can cause minor synaptopathy. However, developmental CHL does not appreciably alter peripheral frequency tuning.

## Introduction

Detection and identification of speech and other sounds in noisy environments is challenging, particularly for hearing-impaired listeners^1,2^. For those with hearing loss, much of this difficulty, known as masking, is assumed to arise from sensorineural damage in the auditory periphery (the cochlea)^3–5^. This is because under sensorineural loss, the acoustic mixture of target and masker abnormally activates the regions of the cochlea encoding the target. Specifically, elevated masking thresholds are ascribed to broadened frequency filters, temporally smeared encoding, and abnormal intensity perception due to cochlear damage, particularly of the outer hair cells^1,3,6–8^. However, even when sensorineural loss is small or absent, perceptual difficulty with masking can occur following *conductive* hearing loss (CHL). For instance, due to middle ear infections (*otitis media),* 5 out of 6 children experience periods of CHL during development^9^, an exposure predicting later problems with speech processing^10,11^. Even after peripheral hearing is restored, children with a history of CHL have increased difficulty with speech in noise perception^12–18^. Thus, there is a timely need to understand the mechanisms by which CHL increases vulnerability to masking.

CHL is broadly used as a model to understand how auditory deprivation affects the central auditory system as it has been assumed to leave the peripheral auditory region intact^19,20^. In addition to attenuating sound energy reaching the cochlea by disrupting sound transmission in the outer or middle ear, CHL can cause changes in the central auditory pathway^21–26^. We have previously suggested that long-term experience with bilateral developmental CHL impairs resilience to masking due to central changes under auditory deprivation^27,28^. A premise for this interpretation was that this CHL should not appreciably alter sensorineural elements in the periphery that could raise masked thresholds. However, this assumption has not been directly tested and is called into question by recent evidence of sensorineural changes to hair cell synapses as a result of extended CHL. Specifically, when tympanic membranes were disrupted in *adult* animals, cochlear histology one year later revealed inner hair cell (IHC) synapse loss, at high frequency regions of the basilar membrane above 5 kHz^29^. In adults, even one month of reversible CHL induced by earplugs was sufficient to induce lasting synapse loss^30^. Synaptic loss is purported to decrease the ability of auditory nerve fibers to encode a masked target^29^, which could have contributed to increased vulnerability to masking in our prior work^27,28^. In addition, when induced during development rather than in adulthood, CHL may further increase the likelihood of changes to the periphery that could impact tuning, as the cochlea is not fully mature prior to hearing onset and thus more susceptible to plastic changes^31–34^. For example, peripheral tuning is determined largely by outer hair cells (OHCs), which are innervated by efferent fibers (medial olivocochlear, MOCs)^35^. The auditory deprivation induced by CHL will change efferent feedback onto these OHCs, potentially altering tuning. Although CHL induced in adulthood did not affect OHCs^29^, peripheral tuning has not been examined after extended developmental CHL.

To examine how chronic bilateral developmental CHL alters peripheral processing, we tested gerbils raised with CHL induced prior to hearing onset. Cochlear compound action potentials (CAPs) are sound-evoked responses representing the summed activity of the auditory nerve to suprathreshold sounds^36,37^ that correlate with synaptic loss^38,39^. To assess the damage to afferent synapses between IHCs and auditory nerve fibers, also referred to as cochlear synaptopathy, we measured CAP amplitudes. To determine the sharpness of the cochlear filters, which would be expected if outer hair cell function was diminished by the CHL, we used forward masking tuning curves measured at the round window as a functional assay^40^. We then compared the sharpness of the peripheral masking tuning curves to a corollary of synaptic loss, CAP amplitudes. Our results show limited evidence of IHC synapse damage and confirm our key assumption that cochlear tuning is not appreciably broadened under CHL. This supports our prior hypothesis that perceptual masking deficits resulting from CHL arise primarily from central auditory changes.

## Methods

### Animals

All procedures relating to the maintenance and use of animals were performed in accordance with the relevant guidelines and approved by the Institutional Animal Care and Use Committee at Northeast Ohio Medical University under protocol number 17–04-077. Seventeen Mongolian gerbils (*Meriones unguiculatus*) from multiple litters were raised by breeding pairs and housed in a 12/12 light/dark cycle. The Control and CHL groups contained 8 and 9 animals respectively (Controls: 4 males, 4 females; CHLs: 2 males, 7 females). CHL was induced at postnatal day (P) 11, and peripheral tuning was measured in adulthood, at ages ranging from P82–P243 (see Table 1; mean 152 ± 21 days).

**Table 1:** Dissection-based confirmation of malleus removal or dislocation. Condition of the malleus, incus, stapes and the malleus/incus joint were determined following CAP recordings. The incus and stapes were intact in all animals. No infection occurred in any animal.

Animal	Age	CAP Threshold (dB SPL)					Malleus intact?	Joint of incus/malleus	Incus intact?	Stapes intact?	Infection?
		1 k	2 K	4 K	8 K	16 K					
M1501	P151	60	65	60	65	60	Long arm removed Head dislocated	Intact	+	+	−
F1502	P152	60	70	55	60	55	Head dislocated	Partially missing	+	+	−
F1528	P139	60	55	60	70	55	Long arm removed Head dislocated	Partially missing	+	+	−
F1627	P82	60	65	45	60	50	Head removed	Intact	+	+	−
F1629	P82	70	70	50	65	55	Head removed	Intact	+	+	−
M1626	P83	65	60	45	60	50	Head removed	Intact	+	+	−
F2150	P196	70	70	70	75	60	Long arm removed Head dislocated	Intact	+	+	−
F2074	P242	50	45	40	50	45	Long arm broken Head partly removed	Intact	+	+	−
F2073	P243	50	55	35	60	65	Long arm broken Head intact	Dislocated	+	+	−

### Malleus surgery

To induce CHL, bilateral malleus surgery was performed on gerbil pups at P11, as previously described^23,27^. This surgery resulted in either complete removal or dislocation of the malleus (see Table 1). Gerbil pups were anesthetized with Metofane (methoxyflurane; Medical Development International ^Ltd^, Australia) until the pedal reflex disappeared. A pre-auricular skin incision was made to expose the ear canals, and pars flaccida of the tympanic membrane was visualized and torn. The malleus was removed or dislocated with forceps, and the incision was sealed with cyanoacrylate. The rest of the tympanic membrane (pars tensa) and the ossicles (incus and stapes) remained intact, as verified by later dissection (see Table 1). Control animals were anesthetized with Metofane, a sham incision was made and sealed with cyanoacrylate. Immediately following surgery, both Control and CHL pups received prophylactic injections of Baytril (enrofloxacin; Bayer, USA) to prevent infections (0.45 mg/ml, 5 mg/kg), and were returned to their parents. Pups were weaned at P30 into unisex groups of siblings. Malleus dislocation and intactness of stapes was verified bilaterally via dissection in adulthood, after CAPs were recorded (see Table 1). Animals that developed middle ear infections were excluded from this study (verified upon dissection).

### CAP recordings

CAPs were recorded from the round window in Control and CHL animals in adulthood (> P80). Gerbils were anesthetized with pentobarbital (60 mg/kg, IP), and given supplementary injections (50 mg/kg, IM) hourly to maintain sedation. A single dose of atropine (1 mg/kg, IM) was injected to avoid airway blockage. A 10 mm right ear post auricular incision^41^ was made after a local injection of lidocaine. The neck muscles were bluntly separated and retracted to expose the middle ear space (bulla). The bulla was opened with forceps and the round window niche was exposed. A silver Teflon-insulated ball electrode was positioned at the round window niche, a stainless steel reference needle electrode was placed subcutaneously at the vertex of the head, and a ground electrode was placed in the right leg muscle. Gerbils were positioned on a homeothermic blanket (Harvard Apparatus, MA, USA) and temperature was maintained at 37 °C. CAP recordings were used to determine thresholds at multiple frequencies, assess likely IHC loss and/or synaptopathy, and measure frequency tuning curves, as described below.

### Stimuli

Stimuli were presented in free field, from a calibrated MF1 multi-field magnetic speaker (Tucker-Davis Technologies (TDT), Alachua FL) positioned 3 cm from the right ear canal. To calibrate the speaker output, a ¼-inch free-field microphone (Brüel and Kjær (B&K) 4939, Denmark) was positioned 3 cm from the center of the MF1 speaker, facing the speaker. The microphone output was amplified (B&K 2690-A Nexus conditioning amplifier) and digitized at a sampling frequency of 195.3 kHz (TDT RZ6 auditory signal processor). A 1–30 kHz frequency sweep was presented and recorded using BioSig RZ software (v5.7.2, TDT) to create a calibration file. These calibration files were applied to spectrally flatten the speaker output, ensuring similar presentation levels at all tested frequencies, across a range of output levels from 0 to 115 dB SPL. Calibrated output levels were verified with custom-written Matlab scripts (Mathworks, Natick MA; scripts by S.J. Shanbhag).

**Figure 1:**
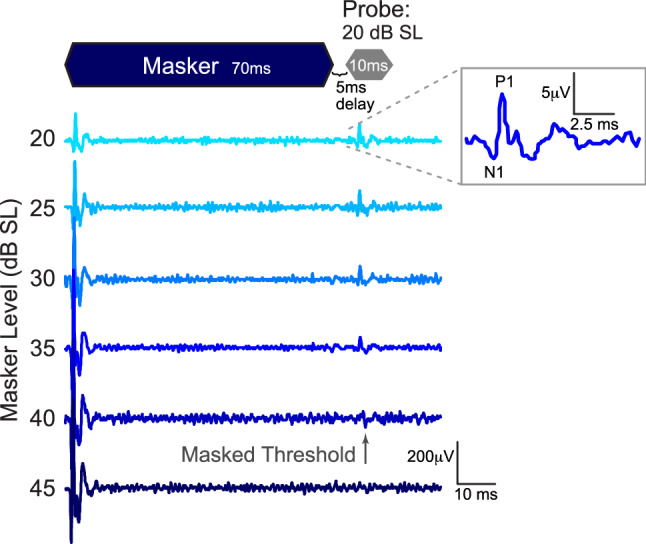
Example of forward masking response used to obtain tuning curves measured at the round window. A two-tone suppression paradigm measured the response to a 10 ms probe tone presented at 20 dB SL. The probe tone was presented 5 ms after the offset of a 70 ms masker tone of a specific frequency (± 1/10 octave steps surrounding the probe tone frequency; here, probe and masker were 4 and 4.8 kHz, respectively). The masker level increased in 5 dB steps; masking threshold was determined as the last visible response to the probe tone. Inset shows the masked response and identifies N1 and P1 peaks.

Probe and masker stimuli were created using SigGen software (v5.7.2, TDT); a schematic is shown in Fig. 1. Probe tones were 10 ms in duration (2 ms rise/fall cosine-squared ramps) with frequencies at 1, 2, 4, 8, and 16 kHz; levels varied from 5 to 105 dB SPL. Masking tones were 70 ms in duration (1 ms rise/fall) presented at varied frequencies and levels. Stimuli were presented at a rate of 8.33/second. All stimuli were presented with BioSigRZ software with alternating polarity to minimize the presence of cochlear microphonics. Traces were averaged across 510 repetitions. Voltage responses were amplified (20 × gain, RA16LI preamplifier, TDT), bandpass filtered (300 Hz to 3 kHz), digitized at 24.4 kHz and 24 bits (RZ6, TDT) and recorded using BioSigRZ on a Dell PC running Microsoft Windows 7.

To determine thresholds and indirectly assess cochlear synaptopathy, we measured the amplitude of wave 1, also known as the N1P1 response, which closely predicts synaptopathy^38,39^. To determine thresholds, we recorded responses to each of the probe tone frequencies without the masker present, with probe tones beginning at loud sound levels (90 and 105 dB SPL for Control and CHL animals, respectively) and decreasing in 5 dB steps until the N1P1 response was no longer visible, as determined by an experienced observer. This point was defined as probe tone threshold for each animal (0 dB SL; Fig. 1). This normalization accounted for a ~ 37 dB shift in probe tone thresholds in CHL animals. We measured the peak-to-peak amplitude of the N1P1 responses to generate CAP input–output functions (measuring response amplitudes to tones varying in level). For 5 animals (n = 2 Control and 3 CHL), 10 dB steps were used, so these animals did not contribute to the averaged functions. Functions were collected at each probe frequency except 1 kHz, where cochlear microphonics prevented clear measurement of peak-to-peak amplitudes. To compare across groups, levels included in the input–output function ranged from each animal’s 0 dB SL threshold to a maximum of 35 dB SL, as the system could not present sounds louder than 35 dB SL for most CHL animals. All animals were tested at least up to 15 dB SL at all frequencies, and most animals were tested up to 35 dB SL at most frequencies.

To determine whether here, CHL compromised masked cochlear frequency tuning in adulthood, we employed a two-tone suppression forward masking paradigm. The probe tone was always presented at 20 dB SL, and 5 ms following masking tone offset. The masking tone frequency was adjusted in ± 1/10 octave steps from the probe tone frequency, and the level was varied until the N1P1 response to the probe tone disappeared^42,43^. The masker level required to eliminate the probe tone response was considered the masked threshold. Masked thresholds were used to create frequency tuning curves.

### Tuning curves

To compare overall shapes of the tuning curves between Control and CHL groups, a linear mixed-effects regression model fitted probe tone thresholds across all masker and probe frequencies. Probe frequency was nested in masker frequency. To adjust for possible sampling variation owing to idiosyncratic effects of individual animals, probe threshold was included as a random effect, in addition to being a covariate. Correlated residual errors accounted for repeated measures within each animal.

Separately, in order to estimate the sharpness of cochlear tuning in a way that allows direct comparison with the human psychophysical literature, raw masked threshold data *W* were fitted using a traditional data fitting approach with a rounded exponential (roex) filter shape^44^:

$$W\left(g\right)=\left(1-r\right)\left(1+pg\right){e}^{-pg}+r$$

The term *p* is an exponential parameter that defines the passband of the filter shape. The term *r* defines the shallow tail portion of the filter outside of the passband. The term *g* is the normalized distance from the center frequency f_C_ of the filter: *g* =|f − f_C_|/f_C_. Using a nonlinear least square solver, *r* and *p* were estimated from the measured *W* as a function of *g* (lsqcurvefit command in Matlab). To quantify the sharpness of the masked tuning curves, at each center probe frequency, Q factors were calculated at the 10 dB down points of the fitted roex functions (e.g., Q_10_ = f_c_/(f_hi(10)_ − f_low(10)_)).

### Statistics

IBM SPSS Statistics 22 (IBM, USA) was used for statistical analysis. CAP thresholds between groups were compared with a two-way repeated-measures mixed analysis of variance (rANOVA), testing for between-subject effects of treatment and within-subject effects of probe frequency. Q values comparing the sharpness of tuning curves were analyzed with a mixed rANOVA. CAP input–output functions were analyzed with a generalized linear mixed regression model, with treatment as a between-subject effect and probe frequency as a within-subject repeated-measures effect. This analysis is robust to missing data, although reduced power due to missing data may increase the chance of a type II error. All *posthoc* tests were adjusted for multiple comparisons using Sidak corrections.

### Experimental design

In summary, CAPs assessed the frequency selectivity of the cochlear response. To measure the threshold shift created by bilateral malleus removal prior to hearing onset, we first recorded CAPs in adulthood at multiple probe tone frequencies. The amplitudes of these responses were used to assess possible synaptopathy, and to determine probe tone thresholds at each frequency. Then, to resolve whether developmental CHL alters peripheral frequency selectivity, we acquired tuning curves using a forward masking paradigm. We fit these curves with an auditory filter model for tonal maskers (a roex function) and used the Q10 factor to quantify and compare the sharpness of tuning. To confirm surgical accuracy and to exclude animals with middle ear infections, a postmortem middle ear dissection was performed in adulthood after measuring CAP thresholds and tuning curves. Malleus absence or dislocation was identified bilaterally, and the intactness of incus and stapes was confirmed. Postmortem dissections were also performed on control animals, to exclude any animals with pathology in the ear canal or middle ear (see Table 1 for details).

## Results

### Threshold shift introduced by CHL

**Figure 2:**
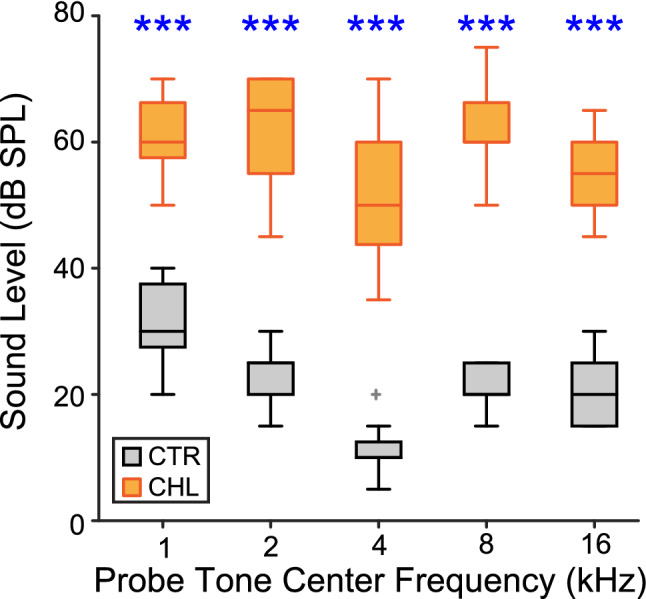
Early malleus displacement induces a hearing loss of ~ 37 dB across a frequency range of 1–16 kHz. CAP thresholds are plotted for each probe frequency, for Controls (CTR) and CHLs (*black and orange*, respectively). Boxplot centers are medians; edges are 25th and 75th percentiles; whiskers extend to the most extreme data points excluding outliers. ***p < 0.0001.

To quantify the CHL introduced by malleus surgery at P11, in adulthood (> P80), CAP thresholds were measured in both Control (n = 8) and CHL animals (n = 9). Thresholds at probe frequencies of 1, 2, 4, 8 and 16 kHz were determined as the lowest sound level that yielded a visible N1P1 response, and are plotted as a function of probe frequency (Fig. 2, *open symbols*). A two-way ANOVA between Controls and CHLs confirmed that the malleus surgery raised thresholds by 30–40 dB at all tested frequencies (main effect of treatment: mean threshold shift across frequencies = 36.77 ± 5.71 dB; F_(1,15)_ = 188.06, *p* < 0.0001; Partial ɳ^2^ = 0.93). Threshold shifts were significant at all individual tested frequencies (Table 2).

**Table 2:** Threshold differences for CHL vs CTR (ANOVA).

Freq (Hz)	Mean diff (dB)	F_(1,15)_	*p*	Partial ɳ^2^
1000	29.3	71.816	0.000*	.827
2000	38.5	126.354	0.000*	.894
4000	39.9	89.616	0.000*	.857
8000	41.5	222.206	0.000*	.937
16,000	34.4	143.845	0.000*	.906

Significance indicated by asterisks.

### Activity-based assessment of synaptopathy

**Figure 3:**
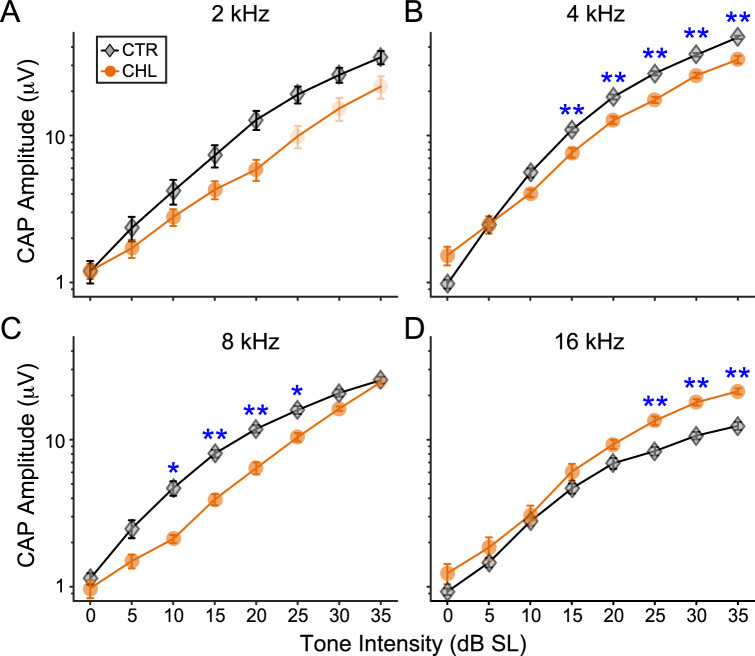
Frequency–response functions at suprathreshold sound levels show evidence for cochlear synaptopathy at some frequencies. (**A**–**D**) Response functions in response to 2, 4, 8 or 16 kHz tones, normalized to threshold for each animal (0 dB SL). Significantly higher or lower responses are indicated by *blue asterisks* (** p < 0.008, * p < 0.02). *Pale symbols* indicate levels with low n’s. Error bars are SEM.

Figure 3 plots wave I peak-to-peak amplitude as a function of SL. CHL affected CAP suprathreshold amplitudes, but did so differently across frequencies (two-way ANOVA between Controls and CHLs, main effect of treatment: F_(1,35)_ = 29.19, *p* < 0.0001; interaction between treatment and probe frequency: F_(1,11)_ = 74.73, *p* < 0.0001). Pairwise comparisons revealed significant differences between the groups at higher signal levels for 4, 8 and 16 kHz (Fig. 3B–D, *blue asterisks*). At 4 and 8 kHz, CHL wave 1 amplitudes were smaller than those of Controls, consistent with cochlear synaptopathy. The same may hold true at 2 kHz, where a lack of significance was potentially due to speaker output limitations which prevented us from collecting more data points at the higher levels (data points where not all animals contributed are depicted by *pale orange symbols*). Similarly, at 8 kHz, only three data points were available at the loudest level tested. At 16 kHz, an unexpected reversal was seen, where CHL wave 1 amplitudes were larger than those of Controls at higher sound levels; no data points were excluded at this frequency.

### Forward masking and tuning curves

**Figure 4:**
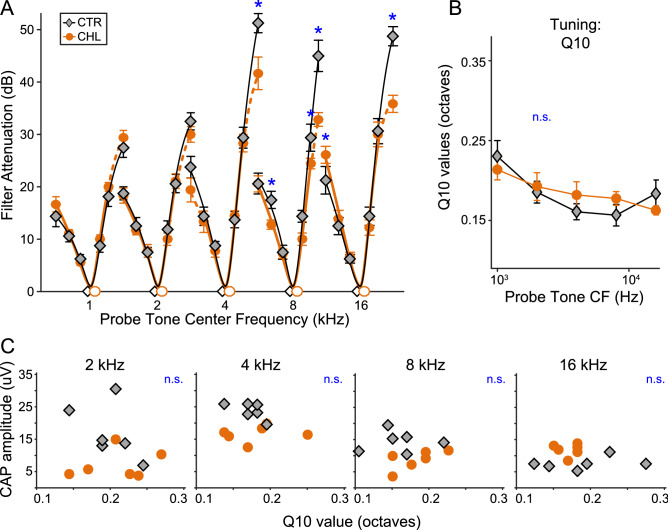
Frequency tuning is not altered by early malleus displacement. (**A**) Forward masked tuning curves were obtained with the probe at 20 dB above threshold, and are depicted normalized to sensation level to allow comparison across treatment groups. Normalized tuning curves were fit using a roex filter function. Masked thresholds are plotted for 1/10 octave frequency steps of maskers surrounding each center probe frequency at 1, 2, 4, 8 and 16 kHz. (**B**) Q_10_ values derived from roex filter functions indicate that conductive hearing loss did not significantly broaden tuning at 10 dB above threshold. (**C**) There are no significant correlations between CAP amplitude and Q10 values for either group at any probe frequency, indicating that synaptopathy does not correlate with cochlear tuning. Error bars are SEM.

To determine whether early-induced CHL compromised cochlear frequency tuning in adulthood, we employed a two-tone suppression forward masking paradigm, measuring masked tuning curves. Boxplots of unnormalized raw values used to create tuning curves are depicted in Fig. 2, and roex-fitted averaged tuning curves, normalized to the center probe tone frequency, are depicted in Fig. 4A. Mean masked tone levels (*closed symbols*) are plotted as a function of their frequencies surrounding each probe tone center frequency (*open symbols*). The curves in Fig. 4A are derived from roex filter fits, allowing comparison of our fits to an established literature^44,45^. This fit assumes that the auditory filter is well fit by a rounded exponential function – i.e., a pair of back-to-back exponentials with a rounded tip, and shallow skirts in the frequency region beyond *∆f/f*_*C*_ = 0.4.

A generalized linear mixed regression on the raw data (normalized to 0 dB SL for each individual) tested the overall shape of tuning, and found no significant effect of treatment group (F_(1,12.58)_ = 0.56, p = 0.47). However, there was a significant effect of masking frequency with nested probe frequency, indicating that tuning curve shapes were not identical across probe frequencies (F_(34,495.4)_ = 138.2, p < 0.0001). Furthermore, there was a significant interaction between treatment group and masking frequency with nested probe frequency, indicating that the curve shapes were altered by CHL treatment (F_(34,495.4)_ = 3.3, p < 0.0001). However, *posthoc* analyses identified that these differences were confined to the flanks of the tuning curves for the center frequencies 4, 8, and 16 kHz (Fig. 4A*, asterisks*; Table 3).

**Table 3:** Forward masking tuning curve statistics (generalized linear mixed regression).

Probe freq (Hz)	Masking freq (Hz)	Mean diff (dB)	Std error	Df	F	*p*
1000	700	2.8	2.193	206.45	1.573	0.211
	800	1.6	2.193	206.45	0.235	0.628
	900	0.2	2.193	206.45	0.008	0.931
	1000	1.3	2.193	206.45	0.353	0.553
	1100	2.4	2.193	206.45	1.176	0.280
	1200	2.8	2.193	206.45	1.624	0.204
	1400	2.0	2.246	218.90	0.777	0.379
2000	1400	0.9	2.172	230.95	0.174	0.677
	1600	0.1	2.172	230.95	0.001	0.972
	1800	0.9	2.172	230.95	0.168	0.682
	2000	1.0	2.172	230.95	0.205	0.651
	2200	1.4	2.172	230.95	0.440	0.508
	2400	1.0	2.172	230.95	0.233	0.630
	2800	2.0	2.409	290.99	0.666	0.415
4000	2800	0.1	2.102	351.82	2.331	0.128
	3200	0.2	2.102	351.82	0.008	0.930
	3600	0.0	2.102	351.82	0.019	0.890
	4000	0.9	2.102	351.82	0.178	0.673
	4400	0.6	2.102	351.82	0.087	0.768
	4800	0.6	2.102	351.82	0.072	0.788
	5600	8.5	2.329	428.90	13.289	0.000*
8000	5600	1.3	2.175	227.00	0.381	0.538
	6400	4.4	2.175	227.00	4.006	0.047*
	7200	0.6	2.175	227.00	0.083	0.774
	8000	1.0	2.175	227.00	0.215	0.643
	8800	3.4	2.175	227.00	2.453	0.119
	9600	5.0	2.175	227.00	5.204	0.023*
	10,400	12.2	2.283	259.62	28.409	0.000*
16,000	11,200	− 5.4	2.139	281.91	6.472	0.011*
	12,800	− 1.8	2.139	281.91	0.677	0.411
	14,400	− 1.5	2.139	281.91	0.490	0.484
	16,000	− 1.0	2.139	281.91	0.221	0.638
	17,600	0.9	2.139	281.91	0.181	0.671
	19,200	0.4	2.139	281.91	0.032	0.859
	22,400	12.4	2.362	359.58	27.695	0.000*

Significance indicated by asterisks.

### Q values and correlations

In addition to comparing the shapes of tuning curves between groups, we calculated the width of individual tuning curves using Q values at 10 dB above the peak of each roex-fitted curve for each subject (mean ± SEM of Q_10_ depicted in Fig. 4B for the two groups). This Q value, a standard measure of neural and perceptual frequency selectivity, indicates the width of tuning curves at 10 dB above threshold. A mixed ANOVA found no treatment effect, indicating that developmental CHL did not appreciably change functional frequency selectivity of the auditory nerve when measured in adulthood (Q_10_: F_(1,15)_ = 0.045, p = 0.824).

Finally, to test whether putative synaptopathy is related to cochlear filter width in either group, we correlated suprathreshold CAP amplitudes (means for each animal from 10 to 35 dB SL) with Q_10_ filter width, across individual animals. There were no significant correlations at any probe frequency for either group (Fig. 4C; Spearman’s rank correlations, p > 0.12 for all comparisons).

## Discussion

Many studies show how reduced peripheral frequency resolution impairs the ability to identify target sound, both in quiet and background noise^46–48^. A hallmark of sensorineural hearing loss, widened peripheral frequency tuning, is considered a key reason for why individuals with sensorineural damage often struggle to understand speech in noisy backgrounds^49^. In contrast, CHL has been assumed to leave peripheral frequency tuning intact, based on assessments of both bottom-up input to the auditory nerve measured via bone conduction thresholds and hair cell counts^19,20^. Despite this, converging evidence shows that CHL (occurring either during development or adulthood) impairs speech comprehension in noise^12–17^. Moreover, using a common animal model that mimics sound deprivation in children with *otitis media* (chronic bilateral CHL, induced prior to hearing onset), our own work shows worsened behavioral tone detection thresholds in both stationary and temporally fluctuating background noise^27,28^. The mechanisms by which CHL increases vulnerability to masking are incompletely understood. We here sought to test the hypothesis that this type of developmental CHL widens peripheral frequency tuning.

The rationale for this study was that the current CHL model may induce synaptopathy and/or broaden tuning, potentially contributing to behavioral deficits that we had previously attributed to changes in the central auditory system. For instance, recent data from aged mice shows loss of IHC synapses following a year of adult-induced CHL^29^, and even following 4 weeks of adult CHL induced by earplugs^30^. This was surprising, because cochlear synaptopathy has been reliably demonstrated in response to excessive sound from noise exposure, not to reduced sound resulting from CHL^38^. Such IHC synaptopathy typically is confined to high threshold fibers which are presumed to support signal in noise processing, as they are resilient to masking by continuous noise^50–52^. Specifically, as background noise increases, these high-threshold fibers are not saturated, so can carry information about relevant sounds^51^. Yet across studies there is inconsistent evidence supporting this idea^39,53^, and a model of > 50% IHC synapse loss does not affect signal in noise processing^54^. Furthermore, peripheral tuning is determined largely by outer hair cells, which are innervated by efferent medial olivocochlear fibers (MOCs)^35^. Efferent activity is reduced during the auditory deprivation induced by CHL, which could affect peripheral tuning. We here induced developmental CHL at hearing onset via malleus dislocation or removal, raising pure tone thresholds by 30–40 dB. Here, reduced CAP N1P1 amplitudes are consistent with mild IHC synaptopathy at 4 and 8 kHz, for sounds above 10 dB SL. However, at 2 kHz, as well as below 15 dB SL, the effect of CHL was too small to reach statistical significance, suggesting that at the lowest tested frequency, CHL does not meaningfully increase synaptopathy. Further at 16 kHz, CAP amplitudes were actually increased by developmental CHL. In addition, the spectral sharpness of peripheral tuning, as assessed by Q_10_, was not affected by CHL at any frequency. The CAP N1P1 amplitudes did not predict filter width at any of the tested frequencies, consistent with a dissociation between functional tuning of the cochlea and synaptopathy. These results are consistent with a milder and transient form of developmental CHL (earplugs inducing ~ 25 dB attenuation), which does not affect peripheral masked tuning curves yet affects perceptual temporal detection tasks^55,56^. Thus, the current data help rule out peripheral confounds to an extensive literature that uses bilateral CHL to study central perceptual effects of auditory deprivation^24,26–28,55,57,58^.

Although CHL did not change the overall shape or sharpness of tuning curves, CHL attenuated the flanks at higher center frequencies (4 – 16 kHz) by 5–10 dB (Fig. 4A, where orange symbols fall below gray symbols only at the edges of the tuning curves). This could be due to ceiling effects, as the maximum output of our system was 115 dB SPL, corresponding to less than 25 dB SL in some of our CHL animals. An acoustically louder stimulus is needed to robustly measure tuning at the flanks in CHL animals, which have a threshold shift of ~ 40 dB. Another possibility is that CHL during development may induce changes in frequency tuning as the cochlea matures, producing an effect due to abnormal development rather than synaptopathy. Prior to maturation, both central and peripheral auditory elements are affected by auditory experience^59–61^. Frequency tuning of the rodent cochlea is still immature at hearing onset, the age when we removed the malleus^31,32,34,62,63^. This immaturity suggests that developmental auditory deprivation could induce plasticity in frequency tuning.

Our data indicate mild IHC synaptopathy at 4 and 8 kHz (Fig. 3). To reconcile the current results with the seemingly contrasting finding of clear synaptopathy from the prior work^29^, two major differences are worth noting across the studies. First, the studies use different species. Unlike mice, gerbils have low frequency hearing, with equivalent low-frequency sensitivity to 250 Hz in gerbils compared with ~ 1000 Hz in mice^64,65^. The previous work tested tones across the range of the most sensitive frequencies in mouse^65^ (~ 5 to 70 kHz) and found synaptopathy only at 8 kHz and higher^29^. Here we similarly tested frequencies across the most sensitive range in the gerbil (from 1 to 16 kHz)^64^, finding modest evidence for synaptopathy at only 4 and 8 kHz. The current data does not address whether CHL may affect tuning or synaptopathy at higher frequencies in gerbils. However, based on mice we would have expected to see an effect across most of the hearing range excluding only the lowest frequencies. Second the previous work tested aged animals, showing that the loss of acoustic drive reduced efferent innervation, a phenomenon thought to exacerbate age-related IHC synaptic loss^29^. With an age range of 2.8 to 8 months, our animals were too young to show age-related hearing loss. In summary, the lack of strong evidence suggesting synaptopathy in our model of developmental CHL as compared to the mouse model with adult-onset CHL may arise from a difference in tested frequencies outside the midrange as compared to the prior work, and/or synaptopathy may be less likely to occur for younger animals, or there could be species differences. It is worth noting that in chinchillas, > 50% loss of the number of IHCs broadened perceptual masked tuning despite leaving tuning in quiet intact^66^. However, CHL in adulthood did not reduce IHC number despite causing IHC synaptopathy^29^. Further studies are needed to assess the effect of extended developmental CHL on IHC number.

### Implications and summary

The current results extend prior work suggesting that central auditory deficits can lead to behavioral hearing deficits, including increased vulnerability to masking, reduced sensitivity to amplitude and frequency modulations, and impaired gap detection^24,26–28,56,57,67^. Long-term CHL weakens inhibitory responses and alters cellular properties throughout the central auditory system^68,69^. In animals with *transient* developmental CHL induced by earplugs from P11–24, peripheral tuning recovers two weeks after removing the earplugs^55^. However, animals with *permanent* developmental malleus removal induced at P11 (as in the current study) display raised peripheral thresholds, amplitudes, and response latencies, as assessed via auditory brainstem responses^24,26^. Importantly, these peripheral effects of CHL do not predict degraded behavioral performance on several perceptual tasks in adulthood, including signal-in-noise detection^24,26,27^. Moreover, in a behavioral assay on a mechanism of masking called modulation masking release, CHL mostly reduced masking mechanisms thought to rely on central rather than peripheral processing^28^. In conjunction, these data indicate that behavioral deficits following CHL arise primarily from central rather than peripheral changes.

In summary, this study addressed whether altered peripheral function introduced a possible confound in prior work suggesting that central changes can degrade behavioral resilience to masking^24,26,27^. The current data show no evidence of appreciable developmental CHL-induced cochlear dysfunction.

## Data Availability

The data are available from the corresponding author on reasonable request.
